# Insights from early experience of a Rare Disease Genomic Medicine Multidisciplinary Team: a qualitative study

**DOI:** 10.1038/ejhg.2017.37

**Published:** 2017-03-22

**Authors:** Elizabeth Ormondroyd, Michael P Mackley, Edward Blair, Jude Craft, Julian C Knight, John Taylor, Jenny C Taylor, Andrew OM Wilkie, Hugh Watkins

**Affiliations:** 1 Division of Cardiovascular Medicine, Radcliffe Department of Medicine, University of Oxford, Oxford, UK; 2 National Institute for Health Research (NIHR) Biomedical Research Centre, Oxford, UK; 3 Wellcome Trust Centre for Human Genetics, University of Oxford, Oxford, UK; 4 Department of Clinical Genetics, Oxford University Hospitals NHS Foundation Trust, Oxford, UK; 5 Oxford NHS Regional Molecular Genetics Laboratory, Oxford University Hospitals NHS Foundation Trust, Oxford, UK; 6 Clinical Genetics Group, Weatherall Institute of Molecular Medicine, University of Oxford, Oxford, UK

## Abstract

Whole-exome/whole-genome sequencing (WES/WGS) has the potential to enhance genetic diagnosis of rare disease, and is increasingly becoming part of routine clinical care in mainstream medicine. Effective translation will require ongoing efforts in a number of areas including: selection of appropriate patients, provision of effective consent, pre- and post-test genetic counselling, improving variant interpretation algorithms and practices, and management of secondary findings including those found incidentally and those actively sought. Allied to this is the need for an effective education programme for all members of clinical teams involved in care of patients with rare disease, as well as to maintain public confidence in the use of these technologies. We established a Genomic Medicine Multidisciplinary Team (GM-MDT) in 2014 to build on the experiences of earlier successful research-based WES/WGS studies, to address these needs and to review results including pertinent and secondary findings. Here we report on a qualitative study of decision-making in the GM-MDT combined with analysis of semi-structured interviews with GM-MDT members. Study findings show that members appreciate the clinical and scientific diversity of the GM-MDT and value it for education and oversight. To date, discussions have focussed on case selection including the extent and interpretation of clinical and family history information required to establish likely monogenic aetiology and inheritance model. Achieving a balance between effective use of WES/WGS – prioritising cases in a diverse and highly complex patient population where WES/WGS will be tractable – and meeting the recruitment targets of a large project is considered challenging.

## Introduction

Whole-exome/whole-genome sequencing (WES/WGS) is rapidly being incorporated into mainstream medicine for enhanced genetic diagnosis of rare disease. WES/WGS is appropriate for individuals and families with a disorder of likely monogenic aetiology.^1^ WES/WGS can be applicable for a wide range of diseases and can discover rare variants in genes known, suspected or not known to be associated with a presenting condition.^2, 3^ In many situations, an accurate genetic diagnosis does not immediately alter treatment or prognosis, but nevertheless, has other clinical and psychosocial benefits for patients and families, such as ending an often protracted diagnostic odyssey by establishing a clear cause for a condition, and identifying family members at risk and who could benefit from surveillance and/or earlier intervention.

The number and selection of the most informative individuals for WES/WGS, as well as the approach taken for data analysis, is determined by the presumed mode of inheritance in the family.^4, 5, 6^ For example, a trio strategy (affected child and unaffected parents) permits more sensitive identification of *de novo* and compound heterozygous mutations if a *de novo* dominant or autosomal recessive condition is suspected.^7, 8, 9, 10^ Autosomal dominant conditions (unless *de novo*) are more difficult to solve; distantly related affected relatives are prioritised for WES/WGS as sharing a smaller proportion of the genome reduces the number of candidate variants, but in small families this number frequently remains high.^3^ There is little published guidance on case selection for WES/WGS or its relationship to outcome, but some initiatives in the US/Canada^11^ and Europe^12^ have recently described implementation of a gate-keeping process.

Interpreting genetic variants is complex, and establishing the clinical validity of a novel candidate variant often requires family segregation studies, testing of unrelated people with similar phenotype and laborious functional studies. Other challenges to the delivery of genomic medicine identified through multidisciplinary collaborations^13, 14^ include lack of institutional and clinical acceptance, limited access to genomics expertise and testing, collection of consistent phenotypic data and capture of dynamic phenotypes, requirement for re-evaluation of genomic data and re-contact, unfamiliarity of patients and healthcare professionals with genetics and the utility of WES/WGS, and handling of genomic secondary findings.^15^ These issues also have an impact on the provision of genetic counselling and consent before and after WES/WGS.

Current practice in the investigation of rare disease in the United Kingdom is being transformed by the 100,000 Genomes Project, which incorporates clinical and research objectives, and has been recruiting through 13 designated Genomic Medicine Centres in the National Health Service (www.genomicsengland.co.uk) since 2015. Individuals and families with rare disease are ascertained through diverse medical specialties after routine clinical assessment and first-line diagnostic genetic testing (gene panel and/or chromosomal microarray).

Several research WES/WGS initiatives in Oxford have preceded the 100000 Genomes Project: WGS500, an initiative begun in 2011 to sequence 500 genomes from patients and family members with diverse rare diseases^3^ was followed by a ‘clinical' WES programme and a separate WGS programme that also aims to sequence 500 genomes. In order to promote effective utilisation of these translational programmes and maximise potential benefit from this significant investment, we established a Genomic Medicine Multidisciplinary Team (GM-MDT) in April 2014. The GM-MDT remit is several fold: in addition to verifying eligibility and approving cases for WES/WGS, providing guidance on recruitment strategy for clinical care teams, and reviewing findings including ‘secondary findings', it promotes genomic education and outreach within the institution. It does not assume responsibility for patient care, which remains with the referring clinician.

We present findings of a study exploring decision-making in the GM-MDT at this pivotal point in translating WES/WGS technology into medical management of rare disease. The aim of the study was to delineate and describe the process of decision-making: the factors that are considered and evaluated by the GM-MDT in reaching decisions. Study findings illustrate how decisions are made both strategically and at an individual case level, and the ways in which local, national and international influences are shaping early genomic medicine practice. This study does not seek to assess the effectiveness of decisions made in terms of outcome, which will be reported when available, together with an analysis of the genetic findings arising from WES/WGS involving the GM-MDT.

## Methods

The study used a qualitative approach: participant observation of GM-MDT meetings, triangulated with semi-structured one-to-one interviews with GM-MDT members. Qualitative methods are ideally suited to understanding complex social processes in context;^16^ qualitative observational methods involve the systematic, detailed observation of behaviour and talk.^17^ In an attempt to minimise the impact on the environment being studied, the researcher may be a ‘participant observer', involved in the activities of the group. The study, part of the wider Molecular Genetic and Clinical studies (MGAC) protocol, was approved by West Midlands Research Ethics Committee, ref. 13/WM/0466.

### Setting

**Figure 1:**
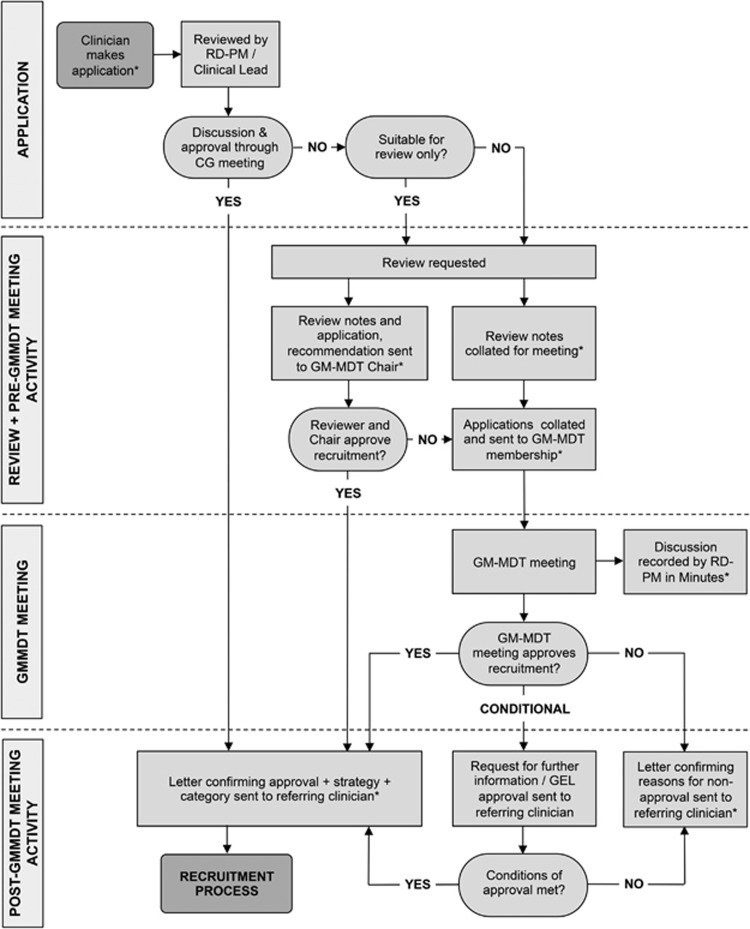
GM-MDT approval process. *Denotes stage at which documents are generated.

Meetings are held monthly on the hospital site and scheduled to last 2 h. During the study period, the number of GM-MDT members ranged from 30 to 39; they were medical doctor/researchers and genetic counsellor/researchers from cardiovascular medicine, clinical genetics, endocrinology, gastroenterology, haematology, immunology, infectious diseases, musculoskeletal diseases, neurology, oncology and renal medicine; clinical (National Health Service) scientists and bioinformaticians; and non-clinical researchers and a project manager. Seventy-five percent of members routinely have patient contact. Membership has changed slightly since inception and attendance is variable. Meetings were quorate when the Chair or deputy, at least four clinical members and at least one laboratory member were present. For WES/WGS case consideration, clinical care teams must submit a short application form summarising clinical information relating to the proband and relatives, pedigree, and preferred sequencing stream. Cases must be submitted in advance of the meeting and are sent for internal peer review by one reviewer with clinical expertise relevant to the case. Cases for review are circulated ahead of the meeting to the team, and briefly presented orally by the reviewer or applicant if present. The applicant is informed in writing of the decision, with any recommendations for further action before acceptance, shortly after the meeting. The approval process is shown in Figure 1.

For WES/WGS programmes under the remit of the GM-MDT, samples were sent for sequencing only after informed, written consent had been obtained. The majority of cases used either an in-house protocol, MGAC (with regard to secondary findings, participants can opt to be informed about medically actionable ‘incidental findings' an amendment approved in March 2015 offers an option of screening of a gene list for ‘additional findings' based on recommendations from the American College of Medical Genetics and Genomics^18^ to participants aged over 16; Supplementary Materials); or 100,000 Genomes Project protocol, which offers consent options for a limited number of additional findings and recessive/X-linked carrier status.

### Data collection

#### Participant observations

Twenty-four meetings (from June 2014, the third meeting, to June 2016; one was missed) were recorded in writing with verbal agreement for the study from the GM-MDT. Meeting notes focused on the dynamic aspects of the meetings, usually the issues generating discussion or debate.

#### Interviews with GM-MDT members

GM-MDT members were approached individually by email between August 2015 and April 2016; thus, 16 meetings had occurred by the time of the first interview. Interviews were conducted by the first author, and lasted between 40 min and 2 h. Written, informed consent was obtained, and interviews were audio-recorded and professionally transcribed verbatim. Interviews are part of a larger study of attitudes towards secondary findings in genomics to be reported elsewhere; only data pertaining to the GM-MDT meetings are included here. Where quotes are included, codes are used in order to protect identities of interviewees.

### Analysis

Thematic analysis was used to analyse written meeting notes and semi-structured interview data.^19^ For analysis of meetings data, an initial coding schema was developed after observation of the first five meetings: what strategic issues are raised; what factors influence case inclusion; what types of further information are deemed necessary; what issues elicit discussion with respect to individual cases and in general. Subsequent meetings were analysed consecutively and iteratively according to this schema by the first and second authors; themes were modified and refined by discussion. NVivo 11 software (QSR International, Melbourne, Victoria, Australia) for qualitative and mixed methods research was used in the analysis of interview data. Interview transcripts were analysed inductively, by open coding and consensus by the first and second authors to generate a coding framework. Coding of interview and observation data, and initial analysis were conducted separately, refined by discussion and then integrated.

## Results

**Figure 2:**
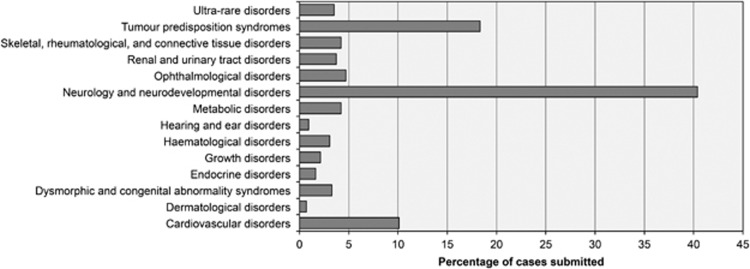
Cases submitted to GM-MDT by speciality.

**Figure 3:**
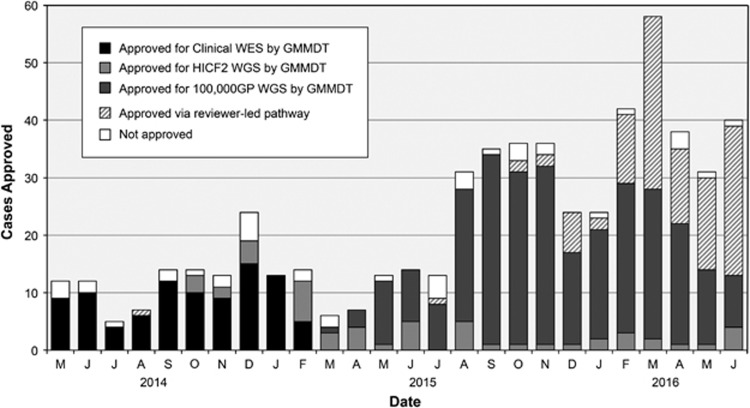
Sequencing stream of cases approved by GM-MDT. HICF2, Health Innovation Challenge Fund; 100,000 GP, Genomics England 100K Genomes Project.

All meetings in the study period were quorate; average attendance was 14 members. Meetings were chaired and followed a written agenda. Policy and project updates and any additional proposed research were presented at the start of the meeting, and results and other business at the end. Case discussion formed the main component of meetings during the study period: between 5 and 34 applications were considered per meeting. A total of 467 cases (cases could be singleton, more than one affected family member, or unaffected parent-trio) were considered across the included meetings, from a range of specialties (Figure 2). Applications for urgent consideration were reviewed by email by two members of the MDT, the Chair plus a specialist in the disease area, and reported at the next scheduled MDT meeting. The study period included active recruitment to the aforementioned programmes: Clinical WES (April 2014–June 2015), Health Innovation Challenge Fund (HICF2) WGS study (April 2014 and ongoing), 100,000 Genomes Project (May 2015 and ongoing). Numbers of cases considered each month—including the number accepted and rejected —are shown in Figure 3. Triaging to the most appropriate programme could be a matter for discussion during MDT meetings. Additional WES/WGS programmes, open to recruitment in Oxford during part or all of the study period—including Deciphering Developmental Disorders, a trio-design WES study for young-onset undiagnosed conditions,^9^ and the NIHR BioResource for Rare Diseases – were not within the remit of the GM-MDT. Meetings frequently overran to allow for discussion of all cases and other business. Internal WES/WGS programmes have been reporting results during the study period; primary findings presented at the GM-MDT have provoked little discussion, results having been discussed with the referring clinician/disease specialist prior to presentation at the GM-MDT. Result presentations were designed to inform and educate on which referrals had been successfully resolved.

A total of 19 members (7 female and 12 male) were interviewed. The full range of professions was represented, and many interviewees had submitted and/or reviewed cases. Members expressed a positive overall impression of the GM-MDT, highlighting the benefits of bringing together specialists in genetics with those from other specialties for the purposes of shared decision-making, increasing the outreach of genomics and education. Four themes were identified: case inclusion for WES/WGS, changing resources, counselling and consent, and group dynamics.

### Case inclusion for WES/WGS

#### Likelihood of an inherited aetiology

The primary consideration in deciding whether a case should be included was likelihood of monogenic causation. Cases prompted discussion of aetiology when the phenotype overlapped with a common condition, such as epilepsy or cancer—the age of the proband at presentation was then a consideration, although it was acknowledged that this can be condition-dependent and the views of relevant specialists were prioritised. Variable expressivity/non-penetrance could also confound apparently congenital presentations such as learning disability. More information on the phenotype was sometimes requested to clarify or rule out non-genetic causes; the extent of phenotypic investigation was sometimes a source of debate, with some members advocating more than had been done in the routine clinical setting. Some clinicians wished to circumvent invasive investigations such as muscle biopsy in a child. Discussions acknowledged that monogenic or even inherited causation could not always be a certainty. During the study period, results from the Deciphering Developmental Disorders study^9^ began to be reported; members who had enrolled cases experienced that apparently monogenic cases were often insoluble by WES. Due to WES-specific limitations, cases unsolved by WES could be considered for subsequent WGS programmes.

#### Mode of inheritance

All WES/WGS programmes under the remit of the GM-MDT target initial analysis to a list of genes reported or suspected to be involved in phenotype causation. MDT discussions were more tolerant of accepting singleton cases if such a gene list could be assembled. Members with experience of genome analysis frequently emphasized the limitations of a singleton approach, expressing the view that this effectively constitutes a panel test. A trio approach, including further samples if available, was preferred when a *de novo* dominant or recessive inheritance pattern was the likely mode of inheritance. When families were consanguineous and large parts of the genome ‘identical by descent', it was considered preferable also to recruit unaffected relatives for genome-wide SNP array typing to allow exclusion of unlinked regions.

Determining the likely inheritance model often requires phenotyping of relatives, and recurring discussions highlighted challenges in establishing affected status:

- When non-penetrance is a possibility, it is unsafe to call asymptomatic individuals ‘unaffected' and assume a *de novo* or recessive model.
- Appropriate clinical testing to rule out a phenotype was condition-specific, may require complex tests and may be of uncertain value. There were debates about how comprehensive phenotyping in relatives should be.
- For conditions with apparent autosomal dominant inheritance – including the major 100,000 Genomes Project disease categories of cancer predisposition and inherited cardiac conditions – phenotyping (and recruiting) geographically remote and elderly relatives was often difficult. Including unaffected relatives – such as an unaffected parent when the other parent was deceased – to discard variants was considered potentially helpful.

#### Prior testing

Exclusion of known or likely genetic causes was a pre-requisite, but gene panels vary widely by disease group according to availability, coverage of implicated genes, reporting time and cost. Options for panel testing, suitability and feasibility were frequently discussed, with the view expressed that WES/WGS programmes should aim to add value to diagnostic testing. The average coverage statistics for these gene panels using WES or WGS was considered to determine the most appropriate test. If several panels might be relevant, the GM-MDT considered it unreasonable that all should be excluded, with the exception of suspected triplet repeat genes. If available panels had a limited reporting rate, long turn-around time or high cost, testing could be waived. A limited panel test performed some time ago need not be repeated. Reassessment of variant pathogenicity taking into account Exome Aggregation Consortium frequencies^20^ or non-segregation occasionally resulted in the ruling out of a panel variant reported as of uncertain significance, and consideration for WES/WGS. Array testing was also frequently requested for complex phenotypes and/or learning disability; occasionally, this identified chromosomal rearrangements that were difficult to interpret in terms of their contribution to phenotype.

#### Clinical utility

The benefits of a genetic diagnosis were clear and pressing in some cases – most frequently childhood onset, severe presentations where parents expressed an intention to use a result for prenatal decision-making, or when treatment might be informed. It was acknowledged that a high level of certainty of pathogenicity would be required in such cases. More commonly, a genetic diagnosis would provide an explanation for the family, reduce the need for further investigations, contribute to genetic counselling about recurrence risk – of enhanced importance in families with consanguineous relationships – or contribute to evaluation of relatives, particularly for later-onset conditions or those with incomplete penetrance.

#### Reasons for declining/deferring cases

The most frequent reason for declining or deferring a case was the requirement for further information: phenotype data relating to the proband, or clinical evaluation and availability of additional family members for recruitment, or family structure. In some cases, further gene or array testing was requested. Occasionally, there was an existing array or gene panel result that was considered potentially explanatory. A small number of cases were declined because they were considered unlikely to be resolved by WES/WGS, usually because family members were unavailable or there was unclear monogenic causation. Clinical utility – although discussed – was rarely a criterion for accepting a case. Interview data show that clinicians who had submitted cases valued the feedback provided (by letter) outlining the reasons for declining a case and whether a re-submission would be considered, although some felt that decisions were not always consistent.

### Changing resources

At the initiation of the GM-MDT, capacity of the two available local WES/WGS programmes was limited by funding. Discussions in early meetings were stringent in selection of cases considered most likely to be tractable to establishing a diagnosis by WES/WGS. As discussion of the impending 100,000 Genomes Project became more frequent (from November 2014, coinciding with Oxford's application to be a Genomic Medicine Centre), it became clear that there would not only be significantly increased capacity for WGS but also an obligation to meet increased recruitment targets. From the point at which 100,000 Genomes Project began recruiting (March 2015), there was a marked increase in case submissions (Figure 3), with a concurrent shift in emphasis towards increasing the volume of applications to meet recruitment targets; the ‘bar becoming lower' was voiced recurrently as a concern that the requirement to meet high targets might have an impact negatively on the quality of applications, in terms of likelihood of successful outcome. Setting the ‘bar' at the right level of stringency in order to enrich for tractable cases while not being too high to exclude appropriate cases was considered important, although this was an intuitive assessment, unverifiable in the absence of data return from the project. Members noted this potential conflict:*So there's always this tension, isn't there, between this fits the criteria for [100,000 Genomes Project] …it ticks all the boxes that we should submit this and actually, this is rubbish science, this is probably not going to give us a result. I think that's a tension within the group. I'm not sure we always get that right. I probably lean more towards doing the right experiment being purist about it but that's probably not realistic always. [G15]*

The point that more comprehensive family work up, phenotyping relatives and recruiting according to the inheritance model would facilitate sequence analysis and have a greater chance of solution was frequently reiterated to maximise value of WES/WGS over panel testing. Interview data concur:*…there's probably such a drive to use up the resources that there are available to complete this work that people are, to some extent, turning a blind eye to the lack of additional phenotypic data which would help the decision-making, and that what's being done is pragmatic. [G13]*

Conversely, others argued that the 100,000 Genomes Project would allow recruitment of multiple unrelated individuals, combined analysis of which would be powerful especially for autosomal dominant disease. This premise accounted for acceptance of WES/WGS cases that remained unsolved through earlier programmes, singletons or re-submission of previously declined cases. There was a coincident perception that the meetings became increasingly pressured with little time for discussion and a concern that they would become unsustainable in their present form. Several members considered that streamlining the process would be required:*As we get used to some of the referrals and they're more routine, there shouldn't be a need to go through this rather convoluted multi-step process…it should be there for exceptional referrals or difficult referrals rather than ones that definitely meet the eligibility criteria and for which there is not much to discuss. [G25]*

Decision-making processes enacted by the GM-MDT were considered to be evolving successfully:*There was a period a few months ago when I felt there was a kind of state of panic amongst the leadership and that actually basic good genetics had slightly gone out of the window in the quest just for sample numbers. But that does seem to have improved, the atmosphere seems to have improved of late, so there's more of a recognition that quality is more important than quantity. [G20]*

Local programmes were favoured if there was a particular clinical imperative, as these programmes were reporting results during the study period, could potentially be accelerated and were interrogable by clinical teams for a progress report. Local programmes were also preferred by some members for cases of research interest, when samples were from overseas collaborators or when similar cases had already been recruited to the same programme; the requirement for individual research teams to maintain a publication record was perceived to factor into decisions to submit cases:*…there is definitely a feeling that people are concerned about what they get back, what they get out of it…Whilst people say they're happy…you can see that sometimes they are thinking…I could have got something personal out of this, which is obviously hard for some clinicians because that's kind of how they attract grant money. They attract support and they build up their reputation. [G26]*

### Counselling/consent

The need to manage patient/family expectations was discussed in the context of specific cases, for example, when the chances of finding a genetic cause were considered low, or in the context of a newly described condition in which understanding was evolving. There was some discussion of the challenges and time required for obtaining informed consent for secondary findings (incidental and/or additional).

### Group dynamics and decision-making

Senior members were most involved in discussions and the decision-making process and were often deferred to; their arguments were often decisive. Interview data show that that this created a somewhat intimidating environment for less senior members, who did not always contribute fully. Many members' attendance was variable, and some saw a potential for inconsistencies in the decisions made:*When there's uncertainty, you need the expertise and if the expertise isn't in the room in that disease area you may get different feedback than if it is… [G28]*

The group collectively decided on the appropriateness of cases for WES or WGS, which occasionally led to a wider debate regarding the ‘grey area' dividing diagnostic and research results. The clinical utility of a screen versus the potential for new disease gene discovery was occasionally discussed. An element of competitiveness was perceived by some, whereas others considered interactions direct, straightforward and honest.

## Discussion

We report on a prospective qualitative study exploring decision-making processes in a novel structure, the GM-MDT, at a pivotal time as WES/WGS moves into routine clinical care. The GM-MDT capitalises on local experience in WES/WGS, predating the 100,000 Genomes Project. Members feel they have benefitted from increased exposure to WES/WGS, opportunities for interaction with people who have diverse clinical and analytic expertise, and value the GM-MDT for the oversight it provides. Of the two main aims of the GM-MDT, case assessment and interpretation of (secondary) findings, the former, specifically the likelihood of monogenic aetiology and recruitment/sequencing strategy, have dominated discussions to date. Competing tensions are apparent in the requirement to fulfil high recruitment targets against strategic recruitment/sequencing that maximises the chances of obtaining a clinically useful result. Some WES/WGS initiatives report a mechanism for consensus input at case consideration,^2, 12^ although many do not. Whether the GM-MDT has in fact impacted on the quality of applications is difficult to assess at this point.

Multidisciplinary teams are an important mechanism for patient clinical management, most frequently studied in relation to management of cancer patients.^21, 22^ An effective MDT ensures collective thinking across disciplines, incorporation of individual views, that evidence-based decisions are reached, and provides for the whole team to contribute.^23^ The GM-MDT is distinct in that it aims to enable effective application of WES/WGS rather than clinical management, and remains an unusual structure in the United Kingdom, although other Centres are developing interdisciplinary structures for review of WGS/WES results (EB, personal communication). Studies of decision-making processes in cancer patient management have found that non-medical/psychosocial issues, which might affect outcome, for example, through adherence to treatment plans, receive less priority. An important role for the GM-MDT envisaged at its inception was in handling of secondary findings; few have been reported to date, as the majority of referrals used a targeted approach in the first instance and few participants had been offered or consented to testing for additional findings. In the GM-MDT, there has been little focus on psychosocial and ethical issues, which might be expected to arise more frequently through identification of secondary findings. It will be important to study decision-making processes around secondary findings, the outcomes of disclosure to patients/families and to ensure a feedback loop such that potential impacts are factored into decisions about which secondary findings are disclosed and how.

The GM-MDT is a resource intensive structure. Some members indicated that they would like to attend meetings more frequently than their schedule allowed, especially since at present WES/WGS represents an additional, rather than a replacement clinical activity. Some suggested that inconsistent attendance might affect decisions taken. During 2015–2016, the number of applications considered per meeting reached a maximum of 34, and discussions were very limited. Members interviewed noted the shift in the content of the meetings and some considered that continuing in the same vein would be unsustainable. This has also limited discussion of results, as the agenda for meetings has largely been preoccupied with applications. From May 2016, applications for 100,000 Genomes Project that were considered unequivocally eligible by the Chair and expert reviewer were approved without MDT discussion (Figure 3).

### Study limitations

Although we have been able to analyse the consensus viewpoint and decisions reached, we cannot analyse or evaluate the process of deliberation, as the meetings were not audio-recorded at the request of some GM-MDT members.

## Conclusions

The GM-MDT builds on and consolidates our experience with WES/WGS since 2011. GM-MDT decisions reported here show that prioritisation of cases likely to be monogenic, appropriate phenotyping of participants – patients and relatives – and recruitment according to likely inheritance model, is considered pre-requisite for successful genetic diagnosis. Moving forward, it will be important to quantify the impact of the GM-MDT's role in approving cases in terms of outcomes, and a detailed presentation of genetic results will be reported elsewhere. However, even at this early stage while such evidence is accumulating, the value of the GM-MDT for educational purposes and raising awareness among diverse healthcare professionals is apparent from the increase in numbers of applications across diverse disease areas, and it is important to ensure that GM-MDT attendance by individuals in genomic medicine across all relevant professions is facilitated.
