# Supplementary material for: Insights from early experience of a Rare Disease Genomic Medicine Multidisciplinary Team: a qualitative study
Source: Eur J Hum Genet. 2017 Mar 22;25(6):680–6. doi: 10.1038/ejhg.2017.37 (PMC5427178; doi:10.1038/ejhg.2017.37)
Supplement: Supplementary Information [file ejhg201737x1.pdf]

Oxford University Hospitals NHS
NHS Trust

UNIVERSITY OF OXFORD

CONSENT FORM

For Patient or relative age 16 and over

Molecular Genetic Analysis and Clinical Studies of Individuals and Families at Risk of Genetic Disease (MGAC)

Contact details of Doctor/Genetic Counsellor/Research Nurse:

Clinic in which recruited:

Genetic condition affecting family:

Patient sticker

Family ID:

If you agree, please initial box

1.	I confirm that I have read and understand the Information Sheet (Version 3.1, 25.02.16) for this study. I have had the opportunity to consider the information, ask questions and have had these answered satisfactorily.	
2.	I understand that my participation is voluntary and I am free to withdraw at any time. This will not affect in any way my present or future medical care and legal rights.	
3.	I agree that study researchers can collect, store and analyse genetic and clinical information, as relevant to the health condition in my family, from my health records. I understand that researchers will keep my information confidential and in a form that protects my identity.	
4.	I understand that data collected during the study may be looked at by authorised individuals from The University of Oxford, OUH NHS Trust, funding agencies and study monitors where it is relevant to my taking part in this research.<br>I permit these individuals to access my research records.	
5.	I agree to provide a sample of blood or saliva, and/or allow samples already collected as part of my medical care to be used for this research.	
6.	I understand and agree that DNA extracted from my sample will be considered a gift to the University of Oxford. If a commercial product were developed as a result of this study, I would not profit financially.	

Depending on the investigations required to pursue a diagnosis for your family's health condition, you may also be asked to choose responses to these statements:

*Please circle decision **and** initial*

7.	I understand that genetic analysis will be performed on DNA extracted from my sample and that **GENES KNOWN TO BE INVOLVED** in the genetic condition affecting my family – if there are any such genes – will be looked at (Phase 1 and 2 analysis).	Yes / No
8.	I understand and agree that, in the event that no disease causing variant is found in initial analysis, or when no such genes are known, **GENES NOT CURRENTLY KNOWN TO BE INVOLVED** in the genetic condition affecting my family will be looked at (Phase 3 analysis).	Yes / No

9. I understand and agree that I will be informed of any results of genetic analysis of my sample, where they ARE relevant to the genetic condition (Pertinent Findings) affecting my family.	Yes / No
10. I understand and agree that I may be informed of any results of genetic analysis of my sample, found **by chance (Incidental Findings),** where they are not relevant to the genetic condition affecting my family but are judged to be important for my/my family's health care, and can be acted upon medically.	Yes / No
11. I agree that researchers may **look in my sample** for variants which are not relevant to the genetic condition affecting my family but are judged to be important for my/my family's health care, and can be acted upon medically (Additional Findings).	Yes / No
12. I agree that confirmed results of any genetic analysis performed and reported via my clinician to me may be shared with other health professionals for the benefit of other members of the family if those family members request it.	Yes / No

Additional - Not part of main study:

1. I agree that my de-identified research data may be deposited in managed databases where it can be accessed on request by other investigators undertaking approved research, in both the public and private sectors, in the UK and internationally.<br>I am free at any time to withdraw my permission for this, but understand that it will not be possible to remove data from research that may already have taken place.	Yes / No
2. I agree to be approached to take part in a sub-study exploring my opinions about this research. I understand that agreeing to be contacted does not oblige me to participate in this sub-study.	Yes / No
3. I agree to be contacted about future ethically approved studies. I understand that agreeing to be contacted does not oblige me to participate in any further studies.	Yes / No

**Name of participant (please print)**	**Signature**	**Date**

In the event of results of analysis becoming available after my death, I would like these individuals to be informed of the results:

Name	Address	Relationship to me

For completion by person taking consent:

I confirm that the purpose of the research, its voluntary nature and its procedures have been explained in terms understandable to this participant.

**Name**	**Signature**	**Date**
